# Dietary Pattern Trajectories from 6 to 12 Months of Age in a Multi-Ethnic Asian Cohort

**DOI:** 10.3390/nu8060365

**Published:** 2016-06-15

**Authors:** Geraldine Huini Lim, Jia Ying Toh, Izzuddin M. Aris, Ai-Ru Chia, Wee Meng Han, Seang Mei Saw, Keith M. Godfrey, Peter D. Gluckman, Yap-Seng Chong, Fabian Yap, Yung Seng Lee, Michael S. Kramer, Mary Foong-Fong Chong

**Affiliations:** 1 Singapore Institute for Clinical Sciences (SICS), Agency for Science, Technology and Research (A*STAR), Singapore 117609, Singapore; gerlimhn@gmail.com (G.H.L.); toh_jia_ying@sics.a-star.edu.sg (J.Y.T.); izzuddin_aris@sics.a-star.edu.sg (I.M.A.); pd.gluckman@auckland.ac.nz (P.D.G.); yap_seng_chong@nuhs.edu.sg (Y.-S.C.); yung_seng_lee@nuhs.edu.sg (Y.S.L.); 2 Department of Obstetrics & Gynaecology, Yong Loo Lin School of Medicine, National University of Singapore, Singapore 119228; chiaairu@u.nus.edu (A.-R.C.); michael.kramer@mcgill.ca (M.S.K.); 3 Department of Nutrition and Dietetics, KK Women’s and Children’s Hospital, Singapore 229899, Singapore; han.wee.meng@kkh.com.sg; 4 Saw Swee Hock School of Public Health, National University of Singapore, Singapore 117549, Singapore; seang_mei_saw@nuhs.edu.sg; 5 Medical Research Council Lifecourse Epidemiology Unit and National Institute for Health Research Southampton Biomedical Research Centre, University of Southampton and University Hospital Southampton National Health Service Foundation Trust, Southampton SO16 6YD, UK; kmg@mrc.soton.ac.uk; 6 Liggins Institute, University of Auckland, Auckland 1023, New Zealand; 7 Department of Paediatrics, KK Women’s and Children’s Hospital, Singapore 229899, Singapore; fabian.yap.kp@kkh.com.sg; 8 Department of Paediatrics, Duke-National University of Singapore Graduate Medical School, Singapore 169857, Singapore; 9 Lee Kong Chian School of Medicine, Nanyang Technological University, Singapore 308232, Singapore; 10 Departments of Paediatrics, Yong Loo Lin School of Medicine, National University of Singapore, Singapore 119228, Singapore; 11 Division of Paediatric Endocrinology and Diabetes, Khoo Teck Puat-National University Children’s Medical Institute, National University Hospital, National University Health System, Singapore 119074, Singapore; 12 Departments of Pediatrics and of Epidemiology, Biostatistics and Occupational Health, McGill University Faculty of Medicine, Montreal, QC H3A 1A2, Canada; 13 Clinical Nutrition Research Centre, Singapore Institute for Clinical Sciences, Agency for Science, Technology and Research, Singapore 117609, Singapore

**Keywords:** infant dietary patterns, dietary pattern trajectories, first year of life, Asian, factor analysis, multilevel mixed models

## Abstract

Little is known about the dietary patterns of Asian infants in the first year of life, nor of their associations with maternal socio-demographic factors. Based on the Growing Up in Singapore towards healthy Outcomes (GUSTO) mother-offspring cohort, cross-sectional dietary patterns were derived by factor analysis using 24-h recalls and food diaries of infants at 6-, 9- and 12-months of age. Dietary pattern trajectories were modeled by mapping similar dietary patterns across each age using multilevel mixed models. Associations with maternal socio-demographic variables, collected through questionnaires during pregnancy, were assessed using general linear models. In *n* = 486 infants, four dietary pattern trajectories were established from 6- to 12-months. *Predominantly breastmilk:* mainly breastmilk and less formula milk, *Guidelines:* rice porridge, vegetables, fruits and low-fat fish and meat, *Easy-to-prepare foods:* infant cereals, juices, cakes and biscuits and *Noodles (in soup) and seafood*: noodle and common accompaniments. In adjusted models, higher maternal education attainment was correlated with higher start scores on *Predominantly breastmilk,* but lowest education attainment increased its adherence over time. Older mothers had higher start scores on *Easy-to-prepare foods*, but younger mothers had increased adherence over time. Chinese mothers had higher start scores on *Predominantly breastmilk* but greater adherence to *Guidelines* over time, while Indian mothers had higher start scores on *Easy-to-prepare foods* but greater adherence to *Predominantly breastmilk* with time (*p* < 0.05 for all). Changes in trajectories over time were small. Hence, dietary patterns established during weaning are strongly influenced by maternal socio-demographic factors and remain stable over the first year of life.

## 1. Introduction

Tracking of eating habits through childhood and into adulthood has been observed in different populations [1,2,3,4], demonstrating the value of healthful dietary patterns early in life in setting the foundation for life-long eating habits [5]. Thus, besides ensuring an adequate supply of key nutrients and calories to achieve optimal growth, development and health during infancy, the establishment of healthful dietary patterns is also critical.

The importance of healthful dietary patterns during infancy is further supported by recent studies demonstrating associations in infant dietary patterns with growth [6,7,8] and cognitive outcomes [9,10]. For example, *Health conscious* and *Family foods* dietary patterns, comprising mainly family table foods, at 9-months of age have been related to lower body mass index (BMI) at the same time point [8], while *Discretionary* dietary pattern trajectories, comprising finger foods that are convenient to prepare, from 6 to 24-months appeared have been associated with lower IQ in adolescence [11]. Most of these studies, however, have examined infancy dietary patterns in Caucasian populations and less is known about dietary patterns in Asian infants.

The influence of maternal socio-demographic factors in shaping infancy dietary patterns is widely recognized. Dietary patterns that adhere to infant feeding guidelines are often adopted by mothers with higher educational levels [6,7,12,13,14,15,16,17], lower BMI [12,13,14], higher household income [7,14,15] and who are multiparous [8,12,14,17,18]. In contrast, unhealthy infant dietary patterns [19] are associated with younger mothers [6,12,13,15,16,17,18,19] and mothers who smoke [6,14,15,17]. The majority of these have been conducted in Caucasian populations [6,8,12,13,14,15,16,17,18,20] with the exception of one study in Japan [18] and another in a multi-ethnic population in America [7]. While it is well established that dietary patterns differ markedly between Asian and Caucasian adults [21,22], the extent to which dietary patterns in Asian infants differ from their Caucasian counterparts and whether maternal predictors of those patterns differ in an Asian infant population remains unexplored.

Weaning represents a transition period during which the diet undergoes rapid changes from milk-based to solid foods, increasing in variety to resemble an adult-like diet. Yet, most studies have limited their scope to a single time-point [7,8,12,17] and may not have adequately accounted for the transitory nature of weaning diets. The concept of modeling dietary patterns longitudinally across time points into dietary trajectories has recently been developed by Smithers *et al.* [11] to address this issue, but no study to date have explored predictors of infant dietary pattern trajectories using this model.

In this study, we aim to better understand the dietary patterns of Asian infants by longitudinally examining the dietary pattern trajectories of infants participating in a multi-ethnic Asian mother-offspring cohort study from 6- to 12-months of age. We also examined potential maternal socio-demographic predictors of these dietary pattern trajectories during this period.

## 2. Subjects and Methods

Our study is based on the Growing Up in Singapore Towards healthy Outcomes (GUSTO) cohort [23]. Between June 2009 and September 2010, pregnant women 18–50 years of age were recruited from Singapore’s two major public maternity units: National University Hospital (NUH) and KK Women’s and Children’s Hospital (KKH). In brief, the participants were Singapore citizens or permanent residents with the intention to deliver in one of the above-named hospitals, reside in Singapore for the next 5 years, spouses were of the same race, and both parents had a homogenous parental background of Chinese, Malay, or Indian ethnicity. The exclusion criteria included receipt of chemotherapy treatment or psychotropic drugs or the presence of serious medical conditions such as type-1 diabetes mellitus. The study received ethical approval from the institutional review boards of the respective hospitals involved. Written consent was obtained from all participants.

### 2.1. Maternal and Infant Characteristics

Socio-demographic characteristics such as maternal age, ethnicity, marital and employment status, housing, monthly household income and education level were obtained from the participants during the first clinic visit upon recruitment (<14 weeks gestation). At 26–28 weeks of gestation, self-reported information on pre-pregnancy BMI, tobacco smoking and alcohol consumption were collected. Details on infant gender and birth order were obtained from birth delivery reports.

### 2.2. Infant Dietary Assessment

Mothers were sent 3-day food diaries to record dietary intakes of their children prior to their postnatal clinic visits at 6-, 9- and 12-months and the diaries were collected at the visits. Instructions and further information on food recording were indicated in the diaries. Mothers who did not complete the food diaries were interviewed by trained personnel at the clinic visits, where a 24-h recall was conducted using the 5-stage, multiple-pass interviewing technique [24]. A set of food pictures and household measurements were provided in the food diaries and during the 24-h recalls to aid in the description of portion size consumed. For the dietary analyses, data from 24-h recalls and 1-day record from the food diaries were used to increase the sample size of the study. Breastmilk consumption via direct breastfeeding was estimated as 780 mL for 6 month-old infants and 600 mL for 9- and 12-month-old infants, based on methods described by Ponza *et al.* [25]. For expressed breastmilk, consumption was quantified according to exact volumes recorded by mothers.

### 2.3. Identifying Dietary Patterns

Food items from the 24-h recalls (*n* = 295, 322, 249 at 6, 9 and 12 months, respectively) or 1-day record (chosen by a randomized order) from the food diaries (*n* = 191, 164, 237 at 6, 9 and 12 months, respectively) were grouped into pre-defined food groups. Food groups with low intakes among the infants (*n* < 3) were further combined based on similarity in culinary usage and nutrient profile [26,27]. A total of 493, 894 and 1137 food items were identified and subsequently grouped into 34, 44 and 61 food groups at 6, 9 and 12 months of age, respectively. Dietary patterns at each time point were extracted by exploratory factor analysis (EFA) using the principal factor method in SPSS version 22.0 (IBM) [28,29] (see Supplementary Materials). EFA solutions were assessed for the magnitude of loadings of food groups, and each pattern was named based on loadings of ≥±0.30 [9,12,13,15,16]. A dietary pattern score, standardized to a mean of zero and standard deviation of one, was calculated for each subject as a function of the contribution (“loading”) that each food made to the pattern. Four dietary patterns were extracted at month-6 and -9 and five dietary patterns at month-12.

### 2.4. Construction of Dietary Pattern Trajectories

Using multi-level mixed models in Stata version 13.0 (Stata Corporation, College Station, Texas, TX, USA), dietary trajectories were empirically constructed by mapping the dietary patterns extracted by EFA at 6-, 9- and 12-months. This procedure accounts for the repeated measures of dietary pattern scores of each subject. The dietary patterns across the three time-points were examined for their similarity in the types of foods and their loadings to ascertain their suitability to be modeled as trajectories. The mapping of each pattern to a trajectory was based on similar key constituent foods (with high loadings) found in the dietary patterns across the three time-points and this correspondingly determined the name of each trajectory [11]. The model generates estimates of intercepts and slopes. The intercept reflects the trajectory score at the start point of each trajectory (6-months of age), while the slope denotes the rate of change in trajectory scores over time. Details have been previously described by Smithers *et al.* [11].

### 2.5. Statistical Analysis

Of the 1247 women recruited, 486 singleton subjects with complete birth measurements and dietary records at 6-, 9- and 12-months were included in the final subgroup (illustrated in Supplementary Materials Figure S1). Pearson’s chi-square tests and independent sample t-tests were used to compare the characteristics of mothers included in this analysis and those with missing dietary records. Pearson’s correlation coefficients were used to assess the correlation between dietary scores derived from 1-day record of the food diaries with the average of the other 2-day records in the subset of mothers who completed the food diaries at the 3 time-points. General linear models were performed to study the associations between dietary trajectory estimates and maternal socio-demographic factors (maternal age, education, ethnicity, employment, BMI at 26-weeks gestation, pre-pregnancy smoking and alcohol status, monthly household income, cohabitation status, parity and infant sex). Socio-demographic factors were stratified into categorical variables and imputations were performed on missing data using the mode. These models were adjusted for potential covariates as described above.

All statistical analyses were performed using the statistical software package SPSS version 22.0 (IBM Corp, New York, NY, USA). A 2-tailed *p*-value of <0.05 was considered to be statistically significant.

## 3. Results

Maternal and infant characteristics of participants with complete dietary records from 6- to 12-months (*n =* 486) were similar to those without complete dietary records (*n* = 438) (Supplementary Materials Table S1). Nonetheless, mothers who provided complete dietary records were more likely to live together with their spouses and had higher monthly household income compared with those who did not provide complete dietary records.

### 3.1. Dietary Pattern Trajectories

**Figure 1:**
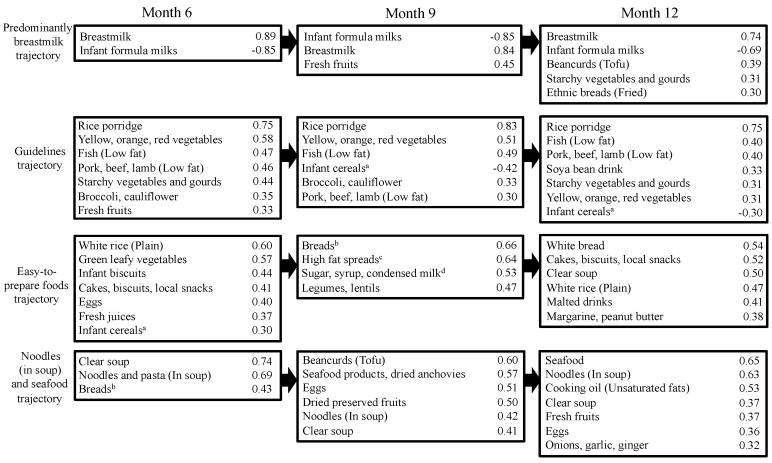
Mapping of dietary patterns at age 6, 9 and 12 months to trajectories. Only foods with loadings ≥±0.30 are shown in the figure. A full list of food items and their loadings are provided in Supplementary Materials. ^a^ Includes rice, wheat and oat baby cereals; ^b^ Includes white and whole wheat breads, breads with fillings or toppings, and baked ethnic breads; ^c^ Includes butter, ghee, peanut butter and margarine; ^d^ Added to drinks and cereals.

Four dietary pattern trajectories were observed from 6- to 12-months and were labeled *Predominantly breastmilk*, *Guidelines*, *Easy-to-prepare foods* and *Noodles (in soup) and seafood* (Figure 1). The *Predominantly breastmilk* trajectory was characterized by breastmilk feeding that exceeded formula milk, the addition of fresh fruits at 9-months, and bean curd (tofu), high-fat ethnic breads and starchy vegetables at 12-months. The *Guidelines* trajectory followed recommended weaning guidelines [30] and was characterized by rice porridge, low-fat fish and meat, a variety of vegetables and fresh fruits as core foods items throughout the 6–12-month period. This trajectory was also characterized by lower intakes of infant cereal at 9- and 12-months.

The *Easy-to-prepare foods* trajectory consisted of foods that require little preparation (infant cereals, juices, cakes and biscuits, as well as adult table foods such as white rice, green vegetables) at 6-months and progressed to easy-to-prepare foods such as breads, spreads, biscuit and confectionaries at 12-months. The *Noodles (in soup) and seafood* trajectory was pre-dominantly noodle-based and consisted of common accompaniments such as eggs, seafood products, dried preserved fruits and bean curd (tofu) found in noodle soup meals. An additional dietary pattern emerged at 12-months of age but did not fall into any of the dietary trajectories: the *Pulses and grains* pattern. This pattern was distinguished by high intakes of nuts and seeds, grains, legumes and lentils and high energy-dense confectionaries. A complete list of food items and their loading scores for each dietary pattern and trajectory is provided in the supplementary file (Supplementary Materials Tables S2–S4). Examples of foods consumed in each item are also provided in Supplementary Materials Table S5. Dietary patterns scores derived from 1-day record of the food diaries were found to be significantly correlated with the other 2-days of the same diaries (correlation coefficient ranged from 0.43 to 0.82) for all dietary patterns and all time points except for the *Easy-to-prepare foods* dietary pattern at 9-months (correlation coefficient = 0.123) (Supplementary Materials Tables S6).

### 3.2. Characteristics of Study Population with Trajectory Estimates

The dietary pattern trajectory estimates of 486 infants summarized with respect to their maternal and infant characteristics are shown in Supplementary Materials Tables S7 and S8. Ranges of intercepts and slopes are listed in the Supplementary Materials. In general, the slopes were small relative to the intercepts.

### 3.3. Associations with Trajectory Intercepts

The fully adjusted associations between the intercepts and maternal socio-demographic characteristics are shown in Table 1. Higher *Predominantly breastmilk* trajectory start scores for infants at 6 months were significantly associated with higher maternal education, higher income, Chinese ethnicity, not working outside the home and having more than one child at home. Higher *Guidelines* trajectory intercepts at 6 months were associated with post-secondary education. Male infants had higher *Easy-to-prepare foods* trajectory intercepts at 6 months, while their mothers were more likely to be older (>34 years old) and of Indian ethnicity. No significant maternal factors were associated with the *Noodles (in soup) and seafood* trajectory.

### 3.4. Associations with Trajectory Slopes

The fully adjusted associations between maternal socio-demographic characteristics and rates of change in dietary trajectory scores (slopes) over the period of 6 to 12-months are shown in Table 2. Mothers of Indian ethnicity, of lower education qualification and primiparous had higher positive slopes for the *Predominantly breastmilk* trajectory. Chinese infants had higher positive slopes for the *Guidelines* trajectory than Malay or Indian infants. Infants with higher slopes for the *Easy-to-prepare foods* trajectory had younger mothers. Chinese infants had higher slopes for the *Noodles (in soup) and seafood* trajectory.

The *Pulses and grain* dietary pattern emerged only at 12-months of age and was associated with Indian ethnicity and mothers with post-secondary education (Supplementary Materials Table S9).

## 4. Discussion

Using multi-level modeling, we integrated food intakes at 6-, 9- and 12-months of age and identified four well-defined dietary patterns and their trajectories over that time period. A key observation was that the rates of change (slopes) of the trajectory scores were much smaller than the differences in the start scores (intercepts) in groups defined by maternal socio-demographic characteristics. This suggests that the maternal characteristics that predict dietary patterns at 6-months of age also largely determine the adherence to the same dietary patterns trajectories for the first year of life. To our knowledge, this is the first study to examine these dietary patterns and associations in an Asian population.

### 4.1. Dietary Patterns

In our study, the *Predominantly breastmilk* pattern resembles the “breastfeeding” pattern and “longer breastfeeding, late contemporary food introduction and use of home-made foods” reported in the ALSPAC [12] and EDEN studies [8,13], respectively. This pattern was characterized by higher intake of breastmilk and lower intake of formula milk and was accompanied by intakes of fruits and vegetables. The *Guidelines* pattern in our study corresponds to the healthy/prudent eating pattern [7,8,14,15,16,17,20] and was characterized by higher intakes of fruits, vegetables, whole grains, poultry and fish. While continued breastfeeding is encouraged during complementary feeding (6–12 months of age), the emphasis in current guidelines for this period is to offer infants a variety of food groups, rather than the type of milk taken [31,32]. The *Easy-to-prepare foods* pattern corresponds to an unhealthy eating pattern characterized by intakes of sugary desserts, high-fat foods and refined grains [6,15,17,18]. Lastly, the *Noodles (in soup) and seafood* pattern, which has not been observed in other studies, appears to reflect Asian adult eating patterns. It resembles the “use of adults’ foods” in the EDEN study [8,13].

### 4.2. Associations between Maternal Factors and Trajectory Intercepts

The trajectory intercept values obtained from the mixed models represents the start point of each dietary pattern trajectory (at 6-months of age). To our knowledge, only three cohort studies to date have reported results for infant dietary patterns at 6-months of age [7,12,17]. Similar to the ALSPAC study [12], higher start scores of our *Predominantly breastmilk* trajectory at 6-months was associated with higher maternal education attainment. The IFPS II study reported that a higher “formula” dietary pattern (reverse of breastmilk) at 6-months of age was negatively associated with maternal education [7]. The relationship we observed with household income is not surprising, as income is closely linked to educational attainment. Its effect on breastfeeding status tends to attenuate when adjusted for education attainment [33,34]. The relationship we observed between higher start scores on the *Predominantly breastmilk* trajectory and unemployment is supported by evidence from studies showing that women working part-time or unemployed are more likely than their counterparts to breastfeed [35,36,37]. In Singapore, working mothers are entitled to 16 weeks of government-paid maternity leave or 12 weeks of maternity leave depending on citizenship, marriage status and duration of employment, but can choose to consume leave non-consecutively [38]. This may in part explain the lower start scores at 6 months on the *Predominantly breastmilk* trajectory for employed mothers when they return to work. Similarly, we found higher educational attainment to be associated with higher start scores of the *Guidelines* trajectory. Mothers with higher education are probably more aware of recommended practices and guidelines for weaning diets. A similar finding was also observed in the IFPS II study [7].

Maternal ethnicity was strongly associated with the start scores of our diet trajectories. Chinese mothers tended to start their infants on the *Predominantly breastmilk* pattern, while Indian ethnicity was associated with the *Easy-to-prepare foods* pattern. Ethnic differences in dietary patterns has also been observed in the American population, where White and African-American mothers scored higher on “guidelines” and “easy-to-prepare foods” patterns compared to their Hispanic counterparts [7], suggesting the importance of culture on diet and weaning practices.

We observed that higher start scores on the *Easy-to-prepare foods* trajectory were also associated with older mothers. In contrast, previous findings suggest that younger mothers tend to provide their children with more convenient foods, perhaps owing to time constraints [39]. Differences in childcare practices among different populations may explain these contrasting findings. For example, young Singaporean couples tend to live with their parents and leave their infants under the grandparents’ supervision. Those infants are more likely to be fed traditional, home-prepared weaning meals than ready-to-eat meals.

No key maternal determinant emerged in the full model analyses for the *Noodles (in soup) and seafood* trajectory, suggesting it may be less influenced by cultural or socioeconomic factors.

### 4.3. Associations between Maternal Factors and Trajectory Slopes

The trajectory slopes represent the rate of change of each dietary pattern trajectory between 6- and 12-months. The means and standard deviations of the trajectory slopes were small (<0.05 ± 0.10) compared to the intercepts (<0.51 ± 1.04), suggesting that infants tend to maintain their dietary patterns over time. The range of the slopes we observed is similar to that reported by Smithers *et al* [11].

Mothers with lowest educational attainment and primiparous had higher slopes for the *Predominantly breastmilk* trajectory, suggesting a greater adherence to this pattern over time. This may not be ideal as the World Health Organization (WHO) recommends that solid foods be introduced shortly after 6-months of age [31] to ensure that infants obtain adequate micronutrients that breastmilk may not supply in sufficient quantity [30,40]. In addition, the variety of food and food textures provided to infants are believed to facilitate the development of oral motor skills and taste preferences and thereby help build a foundation for healthy eating habits [31,32,41].

Mothers in the youngest age group (18–29) tended to have greater adherence to the *Easy-to-prepare foods* trajectory with time, possibly owing to limited knowledge and experience with infant nutrition [39].

Maternal ethnicity continues to be a prevailing determinant of the slope of the dietary pattern trajectories. We observed that while Indian mothers have greater adherence to the *Predominantly breastmilk* trajectory over time, Chinese mothers adhere more to the *Guidelines* and *Noodles (in soup) and seafood* trajectories over time, reflecting a transition to an increasing pattern of table and adult foods. We also observed a *Pulses and grain* dietary pattern emerging at 12-months, particularly in Indian mothers of higher education status.

### 4.4. Strengths and Limitations

Our study captures the important time points of an infant’s weaning diet and fills an important data gap on early feeding patterns. The modeling of dietary pattern trajectories has enabled us to take a longitudinal approach to assessing the overall diet. Compared to conventional cross-sectional analyses, this technique allows us to observe the introduction of new foods through time and the dietary transition during the weaning period. Furthermore, the representation of dietary pattern scores as intercepts and slopes has enabled us to explore the extent to which changes in diet over time are influenced by socio-demographic variables. While the trajectory intercepts signify the cross-sectional dietary pattern scores at 6-months of age, the slopes illuminate the dynamic changes in dietary patterns over time, which cannot be observed in the cross-sectional studies.

Limitations of our study also merit comment. First, single 24-h recalls or 1-day dietary records may not be a good representative of an infant’s usual diet due to day-to-day variation in intakes. However, we have demonstrated high correlation of dietary pattern scores across the 3-days of the food diaries, reflecting good reproducibility of the single day dietary records. Second, GUSTO participants were not randomly sampled from the general population, with deliberate over-sampling of Malay and Indian mothers, and were recruited from two maternity hospitals in Singapore. However, these recruitment sites are the two largest maternity hospitals in Singapore and consist of both private and subsidized patients.

## 5. Conclusions

We examined trajectories of four distinct dietary patterns across three time points between 6- and 12-months. The trajectories were largely determined at the start points (intercepts), and changes over time (slopes) were small. This suggests the importance of establishing the “right” diet at the start of complementary feeding, as dietary changes are more difficult once habits are established. By understanding the key maternal socio-demographic factors that influence dietary patterns during complementary feeding, health professionals and policy-makers can provide more targeted and culturally-appropriate support and advice for mothers and their infants.

## Figures and Tables

**Table 1:** Adjusted associations between dietary pattern trajectory intercepts and sociodemographic characteristics (*n* = 486) ^a^.

Maternal and Child Characteristics	β (95% CI)			
	Predominantly Breastmilk	Guidelines	Easy-to-Prepare Foods	Noodles (in Soup) and Seafood
*Maternal characteristics*				
**Ethnicity**				
Indian	−0.245 (−0.463, −0.026) *	0.069 (−0.035, 0.173)	0.123 (0.063, 0.184) ***	0.028 (−0.015, 0.072)
Malay	−0.259 (−0.476, −0.042) *	−0.069 (−0.172, 0.034)	0.031 (−0.029, 0.091)	0.028 (−0.015, 0.071)
Chinese	*Reference*	*Reference*	*Reference*	*Reference*
**Maternal Age**				
18–29	0.084 (−0.123, 0.292)	−0.026 (−0.125, 0.073)	−0.088 (−0.145, −0.030) **	0.035 (−0.006, 0.076)
30–34	0.040 (−0.161, 0.241)	−0.006 (−0.102, 0.089)	−0.069 (−0.125, −0.014) *	0.034 (−0.005, 0.074)
>34	*Reference*	*Reference*	*Reference*	*Reference*
**Maternal Education** ^b^				
Primary education	−0.781 (−1.021, −0.541) ***	0.081 (−0.033, 0.195)	−0.025 (−0.091, 0.041)	−0.003 (−0.051, 0.044)
Post-secondary	−0.480 (−0.683, −0.277) ***	0.174 (0.077, 0.270) ***	<0.001 (−0.056, 0.056)	−0.007 (−0.047, 0.033)
University and other	*Reference*	*Reference*	*Reference*	*Reference*
**Household Income (SGD)**				
<1999	−0.441 (−0.746, −0.137) **	−0.065 (−0.21, 0.080)	0.053 (−0.031, 0.137)	−0.013 (−0.073, 0.047)
2000–5999	−0.057 (−0.258, 0.145)	−0.034 (−0.13, 0.061)	0.019 (−0.037, 0.074)	0.029 (−0.010, 0.069)
>6000	*Reference*	*Reference*	*Reference*	*Reference*
**Employment**				
Unemployed	0.273 (0.096, 0.449) **	0.014 (−0.069, 0.098)	0.009 (−0.040, 0.058)	0.019 (−0.016, 0.054)
Employed	*Reference*	*Reference*	*Reference*	*Reference*
**Maternal BMI at 26 weeks** ^c^				
<18.5–24.9	0.126 (−0.093, 0.344)	0.015 (−0.089, 0.119)	0.010 (−0.050, 0.070)	−0.022 (−0.065, 0.022)
25.0–29.9	0.214 (−0.005, 0.433)	0.059 (−0.045, 0.163)	0.015 (−0.046, 0.075)	−0.009 (−0.052, 0.034)
>30.0	*Reference*	*Reference*	*Reference*	*Reference*
**Alcohol** ^d^				
Yes	0.080 (−0.095, 0.255)	0.030 (−0.053, 0.113)	−0.019 (−0.068, 0.029)	0.013 (−0.021, 0.048)
No	*Reference*	*Reference*	*Reference*	*Reference*
**Smoking** ^d^				
Yes	−0.209 (−0.456, 0.038)	−0.005 (−0.123, 0.112)	0.039 (−0.030, 0.107)	−0.010 (−0.059, 0.039)
No	*Reference*	*Reference*	*Reference*	*Reference*
**Cohabitation** ^e^				
Not living together	0.022 (−0.719, 0.762)	0.021 (−0.331, 0.372)	−0.039 (−0.244, 0.165)	0.063 (−0.083, 0.209)
Living together	*Reference*	*Reference*	*Reference*	*Reference*
*Infant characteristics*				
**Gender**				
Female	0.027 (−0.122, 0.176)	0.012 (−0.059, 0.082)	−0.042 (−0.083, −0.001) *	−0.016 (−0.045, 0.013)
Male	*Reference*	*Reference*	*Reference*	*Reference*
**Parity**				
First child	−0.202 (−0.371, −0.033) **	0.014 (−0.066, 0.094)	0.032 (−0.015, 0.079)	0.005 (−0.029, 0.038)
Not first child	*Reference*	*Reference*	*Reference*	*Reference*

Abbreviations: BMI, body mass index. ^a^ Data shown are multivariable linear model β coefficients and their 95% confidence intervals (95% CIs) obtained from general linear models. Trajectory intercepts are the dependent variable for formed trajectories from age 6 to 12 months and each characteristic was assessed with adjustments for the other characteristics (covariates) ^b^ Maternal education categorized as primary and secondary education, post-secondary education, as well as university and others; ^c^ Mother’s BMI recorded at 26 weeks of pregnancy (kg/m^2^); ^d^ Status recorded prior to pregnancy; ^e^ Reflects the marital status; single, separated or divorced mothers as living separately; married mothers as living together. * *p*-value < 0.05; ** *p*-value < 0.01; *** *p*-value < 0.001.

**Table 2:** Adjusted associations between dietary pattern trajectory slopes and sociodemographic characteristics (*n* = 486) ^a^.

Maternal and Child Characteristics	β (95% CI)			
	Predominantly Breastmilk	Guidelines	Easy-to-Prepare Foods	Noodles (in soup) and Seafood
*Maternal characteristics*				
**Ethnicity**				
Indian	0.026 (0.013, 0.040) ***	−0.040 (−0.052, −0.028) ***	0.000 (−0.008, 0.008)	−0.013 (−0.020, −0.005) **
Malay	0.013 (−0.001, 0.026)	−0.014 (−0.026, −0.002) *	0.004 (−0.004, 0.012)	−0.013 (−0.021, −0.006) **
Chinese	*Reference*	*Reference*	*Reference*	*Reference*
**Maternal Age**				
18–29	−8.823×10^−5^ (−0.013, 0.013)	0.005 (−0.007, 0.016)	0.009 (0.001, 0.017) *	−0.005 (−0.012, 0.002)
30–34	0.003 (−0.010, 0.015)	0.007 (−0.004, 0.018)	0.006 (−0.002, 0.013)	−0.003 (−0.010, 0.004)
>34	*Reference*	*Reference*	*Reference*	*Reference*
**Maternal Education** ^b^				
Primary education	0.029 (0.014, 0.044) ***	0.001 (−0.012, 0.015)	0.008 (−0.001, 0.017)	−0.002 (−0.010, 0.007)
Post-secondary	0.013 (0.000, 0.026) *	−0.004 (−0.015, 0.007)	0.003 (−0.004, 0.011)	0.004 (−0.003, 0.011)
University and other	*Reference*	*Reference*	*Reference*	*Reference*
**Household Income (SGD)**				
<1999	0.002 (−0.017, 0.021)	−0.013 (−0.030, 0.004)	−0.001 (−0.013, 0.010)	−0.001 (−0.011, 0.010)
2000–5999	−0.009 (−0.022, 0.004)	−0.004 (−0.015, 0.007)	−0.001 (−0.009, 0.006)	−0.002 (−0.009, 0.005)
>6000	*Reference*	*Reference*	*Reference*	*Reference*
**Employment**				
Unemployed	0.007 (−0.004, 0.018)	0.001 (−0.009, 0.011)	0.001 (−0.005, 0.008)	0.002 (−0.004, 0.008)
Employed	*Reference*	*Reference*	*Reference*	*Reference*
**Maternal BMI at 26 weeks** ^c^				
<18.5–24.9	−0.010 (−0.023, 0.004)	0.003 (−0.009, 0.015)	−0.004 (−0.012, 0.004)	0.003 (−0.004, 0.011)
25.0–29.9	−0.008 (−0.022, 0.006)	−0.007 (−0.020, 0.005)	−0.002 (−0.010, 0.006)	0.001 (−0.006, 0.009)
>30.0	*Reference*	*Reference*	*Reference*	*Reference*
**Alcohol** ^d^				
Yes	−0.002 (−0.013, 0.009)	−0.002 (−0.011, 0.008)	0.001 (−0.006, 0.007)	−0.004 (−0.010, 0.002)
No	*Reference*	*Reference*	*Reference*	*Reference*
**Smoking** ^d^				
Yes	0.005 (−0.010, 0.020)	−0.004 (−0.017, 0.010)	−0.002 (−0.012, 0.007)	−0.001 (−0.009, 0.008)
No	*Reference*	*Reference*	*Reference*	*Reference*
**Cohabitation** ^e^				
Not living together	0.011 (−0.035, 0.058)	−0.002 (−0.042, 0.039)	0.018 (−0.010, 0.045)	−0.013 (−0.038, 0.013)
Living together	*Reference*	*Reference*	*Reference*	*Reference*
*Infant characteristics*				
**Gender**				
Female	−0.003 (−0.012, 0.006)	−0.005 (−0.014, 0.003)	0.004 (−0.002, 0.009)	−0.001 (−0.006, 0.004)
Male	*Reference*	*Reference*	*Reference*	*Reference*
**Parity**				
First child	0.014 (0.004, 0.025) **	0.006 (−0.003, 0.016)	−0.006 (−0.012, 0.001)	−0.004 (−0.010, 0.002)
Not first child	*Reference*	*Reference*	*Reference*	*Reference*

Abbreviations: BMI, body mass index. ^a^ Data shown are multivariable linear model β coefficients and their 95% confidence intervals (95% CIs) obtained from general linear models. Dietary pattern trajectory slopes as the dependent variable for patterns that formed trajectories from age 6 to 12 months and each characteristic was assessed with adjustments for the other characteristics; ^b^ Maternal education categorized as primary and secondary education, post-secondary education, as well as university and others; ^c^ Mother’s BMI recorded at 26 weeks of pregnancy (kg/m^2^); ^d^ Status recorded prior to pregnancy; ^e^ Reflects the marital status; single, separated or divorced mothers as living separately; married mothers as living together. * *p*-value < 0.05; ** *p*-value < 0.01; *** *p*-value < 0.001.

## Supplementary Materials

The following are available online at http://www.mdpi.com/2072-6643/8/6/365/s1, Figure S1: Flowchart of selection process. Only subjects with complete dietary records from 6 to 12 months were included in the analysis (*n* = 486), Table S1: Comparisons of characteristics between study group and those excluded due to incomplete dietary records from 6 to 12 months, Table S2: Varimax-rotated component matrix loadings of food items on four dietary patterns extracted by EFA at 6 months of age (*n* = 486), Table S3: Varimax-rotated component matrix loadings of food items on four dietary patterns extracted by EFA at 9 months of age (*n* = 486), Table S4: Varimax-rotated component matrix loadings of food items on four dietary patterns extracted by EFA at 12 months of age (*n* = 486), Table S5: Examples of foods consumed under each food item at 6, 9 and 12 months of age, Table S6: Correlation coefficients for dietary pattern scores derived from 1-day record and average of 2-day record of the food diaries, Table S7: Dietary pattern trajectory intercepts and slopes of infants according to characteristics of study sample (*n =* 486), Table S8: Dietary pattern trajectory intercepts and slopes of infants according to characteristics of study sample (*n =* 486), Table S9: Associations between “Pulses and grains” dietary pattern scores at 12 months and sociodemographic characteristics (*n* = 486).
