# Supplementary material for: Dietary Pattern Trajectories from 6 to 12 Months of Age in a Multi-Ethnic Asian Cohort
Source: Nutrients. 2016 Jun 15;8(6):365. doi: 10.3390/nu8060365 (PMC4924206; doi:10.3390/nu8060365)
Supplement: Supplementary file 1 [file nutrients-08-00365-s001.doc]

Supplementary Materials: Dietary Pattern Trajectories from 6 to 12 Months of Age in a Multi-Ethnic Asian Cohort

Geraldine Huini Lim, Jia Ying Toh, Izzuddin M. Aris, Ai-Ru Chia, Wee Meng Han,
Seang Mei Saw, Keith M. Godfrey, Peter D. Gluckman, Yap-Seng Chong, Fabian Yap,
Yung Seng Lee, Michael S. Kramer and Mary Foong-Fong Chong


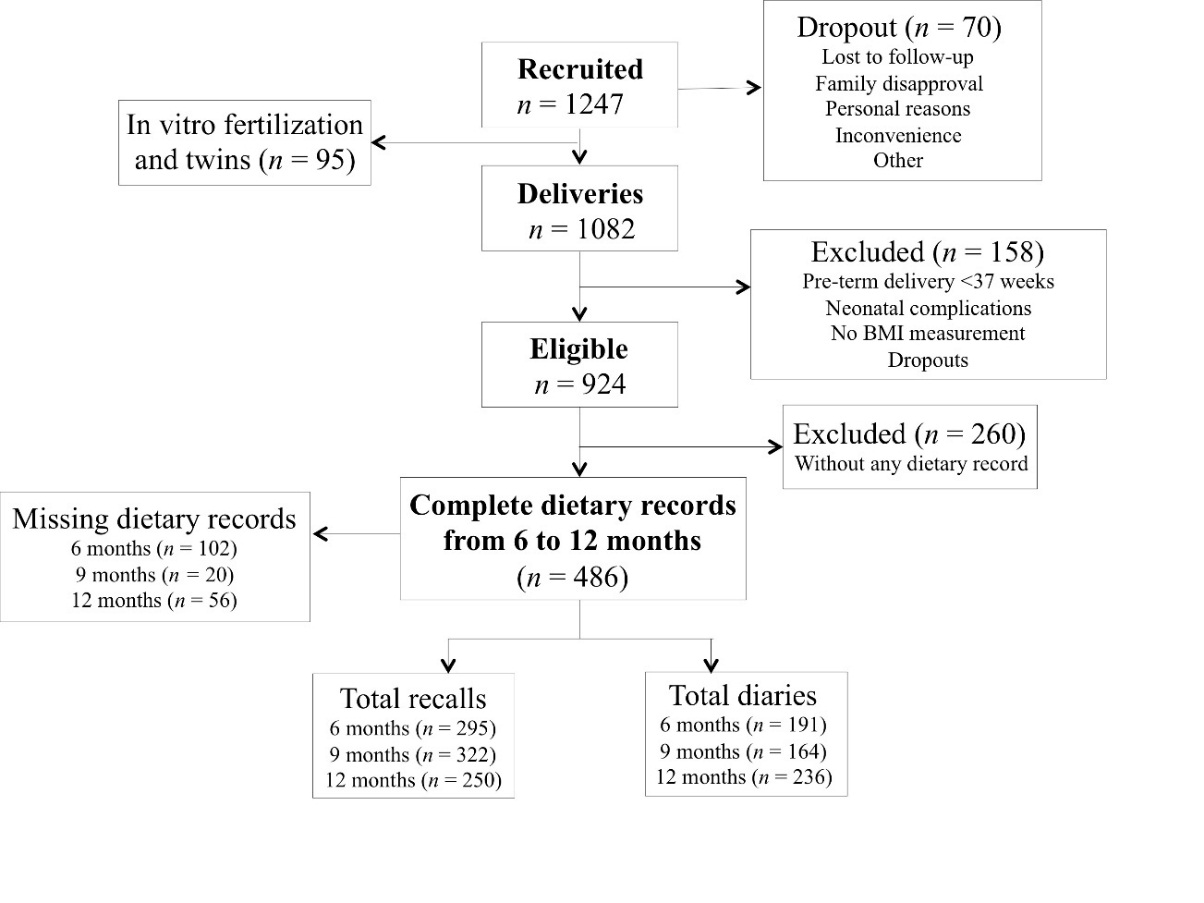


**Figure S1.** Flowchart of selection process. Only subjects with complete dietary records from 6 to 12 months were included in the analysis (*n* = 486).

**Table S1.** Comparisons of characteristics between study group and those excluded due to incomplete dietary records from 6 to 12 months.

| **Maternal & Child Characteristics** | **Study Group (*n* = 486)** | | **Incomplete Dietary  Records (*n* = 438)** | | ***p*-Value ^c^** |
| --- | --- | --- | --- | --- | --- |
|  | **Mean/n ^a^** | **SD/% ^b^** | **Mean/n** | **SD/%** |  |
| *Maternal characteristics* |  |  |  |  |  |
| **Ethnicity** |  |  |  |  | 0.17 |
| Indian | 83 | 17.1 | 90 | 20.5 |  |
| Malay | 121 | 24.9 | 120 | 27.4 |  |
| Chinese | 282 | 58.0 | 228 | 52.1 |  |
| **Maternal Age** |  |  |  |  | 0.52 |
| 18–29 | 206 | 45.7 | 200 | 42.4 |  |
| 30–34 | 169 | 31.5 | 138 | 34.8 |  |
| >34 | 111 | 22.8 | 100 | 22.8 |  |
| **Maternal Education** ^d^ |  |  |  |  | 0.33 |
| Primary education | 137 | 28.2 | 141 | 32.2 |  |
| Post-secondary | 174 | 35.8 | 156 | 35.6 |  |
| University and other | 175 | 36.0 | 141 | 32.2 |  |
| **Household Income (SGD)** |  |  |  |  | 0.02 |
| <1999 | 62 | 12.8 | 65 | 14.8 |  |
| 2000–5999 | 274 | 56.4 | 273 | 62.3 |  |
| >6000 | 150 | 30.9 | 100 | 22.8 |  |
| **Employment** |  |  |  |  | 0.30 |
| Unemployed | 147 | 30.2 | 119 | 27.2 |  |
| Employed | 339 | 69.8 | 319 | 72.8 |  |

***Table S1.*** *Cont.*

| **Maternal & Child Characteristics** | **Study Group (*n* = 486)** | | **Incomplete Dietary  Records (*n* = 438)** | | ***p*-Value ^c^** |
| --- | --- | --- | --- | --- | --- |
|  | **Mean/n ^a^** | **SD/% ^b^** | **Mean/n** | **SD/%** |  |
| **Maternal BMI at 26 weeks** ^e^ |  |  |  |  | 0.68 |
| <18.5–24.9 | 219 | 45.1 | 206 | 47.0 |  |
| 25.0–29.9 | 180 | 37.0 | 150 | 34.2 |  |
| >30.0 | 87 | 17.9 | 82 | 18.7 |  |
| **Alcohol** ^f^ |  |  |  |  | 0.21 |
| Yes | 171 | 35.2 | 137 | 31.3 |  |
| No | 315 | 64.8 | 301 | 68.7 |  |
| **Smoking** ^f^ |  |  |  |  | 0.77 |
| Yes | 59 | 12.1 | 56 | 12.8 |  |
| No | 427 | 87.9 | 382 | 87.2 |  |
| **Cohabitation** ^g^ |  |  |  |  | 0.02 |
| Not living together | 5 | 1.0 | 14 | 3.2 |  |
| Living together | 481 | 99.0 | 424 | 96.8 |  |
| *Infant characteristics* |  |  |  |  |  |
| **Gender** |  |  |  |  | 0.84 |
| Female | 232 | 47.7 | 212 | 48.4 |  |
| Male | 254 | 52.3 | 226 | 51.6 |  |
| **Parity** |  |  |  |  | 0.86 |
| First child | 207 | 43.2 | 189 | 43.2 |  |
| Not first child | 279 | 56.8 | 249 | 56.8 |  |
| z-BMI at birth | −0.2 | 1.0 | −0.2 | 1.0 | 0.70 |

Abbreviations: BMI, body mass index. ^a^ Reflects mean of continuous variables or frequency for categorical variables; ^b^ Reflects standard deviation of continuous variables or percentages of categorical variables; ^c^ Results shown are *p*-values obtained from pearson chi-square test for categorical variables and independent samples *t*-test for continuous variables; ^d^ Maternal education categorized as primary and secondary education, post-secondary education, as well as university and others; ^e^ Mother’s BMI recorded at 26 weeks of pregnancy (kg/m^2^); ^f^ Status recorded prior to pregnancy; ^g^ Reflects the marital status; single, separated or divorced mothers as living separately; married mothers as living together.

**Table S2.** Varimax-rotated component matrix loadings of food items on four dietary patterns extracted by EFA at 6 months of age (*n* = 486).

| **Food Items** | **Dietary Patterns** | | | |
| --- | --- | --- | --- | --- |
|  | **Guidelines** | **Predominantly  Breastmilk** | **Easy-to-Prepare  Foods** | **Noodles (in Soup)  and Seafood** |
| Rice porridge | **0.75** | 0.00 | 0.04 | 0.11 |
| Yellow, orange, red vegetables | **0.58** | −0.06 | 0.05 | 0.15 |
| Fish, (Low fat) | **0.47** | −0.04 | −0.09 | 0.26 |
| Pork, beef, lamb (Low fat) | **0.46** | 0.14 | 0.13 | −0.08 |
| Starchy vegetables and gourds | **0.44** | 0.01 | −0.06 | 0.03 |
| Broccoli, cauliflower | **0.35** | −0.05 | −0.04 | −0.18 |
| Fresh fruits | **0.33** | 0.20 | 0.14 | −0.06 |
| Dried preserved fruits | 0.20 | 0.01 | −0.08 | 0.01 |
| Seafood products, dried anchovies | 0.18 | 0.07 | 0.08 | −0.03 |
| Brown rice (Plain) | 0.17 | 0.02 | −0.09 | −0.12 |
| Other grains ^a^ | 0.14 | 0.14 | 0.09 | −0.10 |
| Onions, garlic, ginger | 0.11 | 0.01 | 0.06 | −0.04 |
| Breastmilk | −0.09 | **0.89** | −0.16 | 0.01 |
| Infant formula milks | 0.04 | −**0.85** | 0.09 | −0.01 |
| Infant bottled products | −0.14 | −0.20 | 0.01 | −0.02 |
| Sweetened drinks | −0.08 | −0.14 | 0.00 | 0.14 |
| Oats porridge | 0.09 | 0.13 | −0.10 | −0.09 |
| Traditional drinks ^b^ | 0.00 | −0.11 | −0.05 | 0.04 |
| White rice (Plain) | −0.09 | 0.17 | **0.60** | 0.26 |
| Green leafy vegetables | 0.16 | 0.23 | **0.57** | 0.17 |
| Water | 0.10 | −0.23 | **0.45** | −0.14 |
| Infant biscuits | −0.14 | −0.15 | **0.44** | 0.04 |

***Table S2.*** Cont.

| **Food Items** | **Dietary Patterns** | | | |
| --- | --- | --- | --- | --- |
|  | **Guidelines** | **Predominantly  Breastmilk** | **Easy-to-Prepare  Foods** | **Noodles (in Soup)  and Seafood** |
| Cakes, biscuits, local snacks | 0.00 | **0.32** | **0.41** | 0.20 |
| Eggs | 0.10 | 0.01 | **0.40** | −0.14 |
| Fresh juices | −0.09 | −0.08 | **0.37** | 0.04 |
| Infant cereals | −0.13 | −0.21 | **0.30** | −0.24 |
| Legumes and pulses | 0.06 | 0.13 | 0.14 | 0.07 |
| Dairy products | 0.06 | 0.00 | 0.12 | −0.11 |
| Nuts, oils, ghee | 0.08 | 0.08 | 0.11 | −0.01 |
| Sugar confectioneries | −0.03 | −0.01 | −0.03 | 0.01 |
| Clear soup | 0.02 | 0.00 | −0.05 | **0.74** |
| Noodles and pasta (in soup) | 0.15 | −0.13 | −0.13 | **0.69** |
| Breads ^c^ | −0.08 | 0.12 | 0.10 | **0.43** |
| Poultry (Low fat) | 0.01 | −0.13 | 0.09 | 0.26 |

^a^ Includes ragi flour, millet and sweetcorn; ^b^ Traditional drinks include barley water and chrysanthemum water; ^c^ Includes white and wholewheat, breads with fillings or toppings, and baked ethnic breads.

**Table S3.** Varimax-rotated component matrix loadings of food items on four dietary patterns extracted by EFA at 9 months of age (*n* = 486).

| **Food Items** | **Dietary Patterns** | | | |
| --- | --- | --- | --- | --- |
|  | **Guidelines** | **Predominantly Breastmilk** | **Noodles (in Soup) And Seafood** | **Easy-to-Prepare Foods** |
| Rice porridge | 0.83 | −0.01 | 0.02 | 0.07 |
| Yellow, orange, red vegetables | 0.51 | 0.09 | −0.18 | 0.03 |
| Fish (Low fat) | 0.49 | 0.14 | −0.04 | 0.07 |
| Infant cereals | −0.42 | −0.02 | −0.04 | −0.04 |
| Broccoli, cauliflower | 0.33 | 0.23 | −0.04 | −0.05 |
| Pork, beef, lamb (Low fat) | 0.30 | −0.06 | 0.10 | −0.18 |
| Poultry ^a^ | 0.25 | −0.05 | −0.10 | 0.00 |
| Starchy vegetables and gourds | 0.23 | 0.14 | −0.08 | −0.09 |
| Green leafy vegetables | 0.21 | 0.20 | 0.02 | 0.02 |
| Fresh juices | 0.17 | −0.09 | −0.13 | −0.12 |
| Oats porridge | 0.16 | 0.00 | 0.03 | 0.00 |
| Infant bottled products | −0.12 | −0.12 | −0.10 | −0.06 |
| Infant formula milks | −0.01 | −0.85 | 0.00 | −0.10 |
| Breastmilk | −0.02 | 0.84 | −0.06 | 0.05 |
| Fresh fruits | 0.06 | 0.45 | 0.08 | 0.04 |
| Pasta | 0.07 | 0.15 | −0.05 | −0.04 |
| Brown rice (Plain) | 0.04 | 0.15 | −0.05 | −0.06 |
| Yoghurt and cultured drinks | −0.09 | 0.15 | −0.01 | 0.01 |
| Indian breads (Fried) | −0.12 | 0.13 | −0.02 | 0.06 |
| Sauces and others | −0.07 | −0.09 | −0.06 | −0.05 |
| Flavoured rice | 0.02 | −0.09 | −0.02 | 0.08 |
| Milk and malted drinks | 0.00 | 0.09 | 0.03 | −0.03 |
| Bean curds (Tofu) | 0.12 | 0.02 | 0.60 | 0.06 |
| Seafood products and dried anchovies | 0.28 | −0.08 | 0.57 | 0.08 |
| Eggs | −0.06 | −0.09 | 0.51 | 0.02 |
| Dried preserved fruits | 0.19 | −0.04 | 0.50 | 0.05 |
| Noodles (in soup) | −0.19 | 0.07 | 0.42 | −0.19 |
| Clear soup | −0.13 | 0.12 | 0.41 | −0.20 |
| Sweetened drinks | 0.22 | −0.11 | 0.26 | 0.06 |
| Sweet spreads and others | −0.08 | 0.13 | 0.24 | 0.04 |
| Infant biscuits | 0.03 | 0.07 | −0.18 | 0.04 |
| Other grains ^b^ | −0.01 | 0.03 | 0.17 | −0.04 |
| Cakes, biscuits, local snacks | −0.02 | −0.09 | 0.13 | 0.00 |
| Nuts and oils | −0.05 | 0.04 | 0.09 | 0.00 |
| Commercial breads ^c^ | 0.00 | −0.12 | 0.03 | 0.66 |
| High fat spreads ^d^ | −0.01 | 0.00 | −0.02 | 0.64 |
| Sugar, syrup, condensed milk | −0.09 | −0.12 | 0.00 | 0.53 |
| Legumes, lentils | −0.10 | 0.18 | −0.02 | 0.47 |
| Water | 0.16 | −0.15 | −0.02 | 0.23 |

***Table S3.*** *Cont.*

| **Food Items** | **Dietary Patterns** | | | |
| --- | --- | --- | --- | --- |
|  | **Guidelines** | **Predominantly Breastmilk** | **Noodles (in Soup) And Seafood** | **Easy-to-Prepare Foods** |
| Cheese | 0.15 | −0.05 | −0.07 | 0.20 |
| White rice (Plain) | −0.15 | 0.06 | −0.01 | 0.17 |
| Onions, garlic, ginger | 0.02 | 0.12 | −0.03 | 0.12 |
| Traditional drinks ^e^ | 0.02 | −0.07 | −0.04 | −0.08 |
| Dim sums | −0.04 | −0.06 | 0.03 | −0.07 |

^a^ Includes all poultry prepared using any cooking method; ^b^ Includes sweetcorn, baby corn, millet, barley, ragi and wheat flour, rice and semolina powder; ^c^ Includes white breads, wholemeal breads and breads with fillings or toppings; ^d^ Includes butter, ghee, peanut butter and margarine; ^e^ Includes barley, herbal and red date water.

**Table S4.** Varimax-rotated component matrix loadings of food items on four dietary patterns extracted by EFA at 12 months of age (*n* = 486).

| **Food Items** | **Dietary Patterns** | | | | |
| --- | --- | --- | --- | --- | --- |
|  | **Pulses and  Grains** | **Guidelines** | **Predominantly Breastmilk** | **Easy-to-Prepare Foods** | **Noodles (in Soup) and Seafood** |
| Nuts and seeds | **0.69** | 0.13 | −0.06 | −0.06 | 0.08 |
| Sugar, syrup, condensed milk | **0.53** | 0.02 | −0.02 | **0.32** | 0.01 |
| Other grains ^a^ | **0.51** | 0.05 | 0.08 | −0.09 | 0.19 |
| Butter, ghee | **0.43** | 0.07 | −0.02 | 0.02 | −0.07 |
| Legumes, lentils | **0.43** | −0.17 | 0.21 | 0.03 | −0.02 |
| Yellow, orange, red vegetables | **0.39** | **0.31** | −0.08 | −0.02 | −0.05 |
| Cheese | 0.21 | −0.05 | 0.11 | −0.07 | −0.09 |
| Breakfast cereals | −0.14 | 0.13 | 0.10 | 0.11 | −0.04 |
| Fish and seafood products | −0.13 | 0.01 | 0.07 | 0.09 | 0.06 |
| Sweet snacks | −0.04 | −0.03 | −0.02 | −0.01 | −0.03 |
| Rice porridge | −0.06 | **0.75** | 0.01 | −0.14 | −0.17 |
| Fish (Low fat) | −0.06 | **0.40** | −0.04 | −0.18 | −0.02 |
| Pork, beef, lamb (Low fat) | −0.11 | **0.40** | 0.03 | −0.07 | 0.02 |
| Soya bean drink | −0.01 | **0.33** | 0.08 | 0.05 | −0.05 |
| Starchy vegetables and gourds | 0.31 | **0.31** | −0.04 | 0.03 | −0.08 |
| Infant cereals | 0.01 | −**0.30** | 0.04 | −0.19 | −0.16 |
| Yoghurt and cultured drinks | 0.24 | −0.28 | 0.22 | 0.01 | 0.12 |
| Green leafy vegetables | −0.15 | 0.27 | 0.00 | −0.01 | 0.12 |
| Potatoes (Fried) | −0.04 | −0.21 | −0.07 | 0.00 | −0.08 |
| Broccoli, cauliflower | 0.10 | 0.19 | 0.04 | −0.05 | 0.00 |
| Wholemeal bread | −0.04 | −0.15 | 0.03 | −0.10 | −0.05 |
| Traditional drinks ^b^ | −0.03 | −0.14 | 0.08 | −0.08 | −0.04 |
| Dried preserved fruits | −0.04 | −0.14 | −0.02 | −0.04 | 0.03 |
| Fresh juices | 0.04 | −0.14 | −0.05 | 0.05 | −0.12 |
| Seafood products, dried anchovies | −0.01 | 0.13 | −0.07 | −0.01 | 0.05 |
| Flavored rice | −0.04 | −0.13 | −0.06 | −0.04 | −0.03 |
| Pasta | −0.08 | −0.11 | 0.06 | −0.02 | 0.11 |
| Oats porridge | −0.07 | 0.09 | 0.05 | 0.00 | −0.04 |
| Sauces and others | −0.05 | −0.09 | 0.00 | 0.03 | 0.03 |
| Breastmilk | 0.09 | 0.01 | **0.74** | −0.08 | 0.08 |
| Infant formula milks | −0.12 | −0.19 | −**0.69** | −0.04 | −0.07 |
| Bean curds (Tofu) | −0.16 | 0.19 | **0.39** | 0.21 | −0.02 |
| Ethnic breads ^c^ (Fried) | 0.18 | −0.29 | **0.30** | −0.06 | −0.06 |
| Water | 0.18 | 0.17 | −0.29 | 0.01 | 0.00 |
| Milk | 0.15 | 0.04 | 0.29 | 0.15 | 0.01 |
| Gravy, curry-based | 0.08 | −0.23 | 0.28 | −0.03 | −0.04 |
| Tea with sugar | −0.16 | 0.08 | 0.23 | 0.19 | −0.02 |
| Infant bottled products | 0.00 | −0.11 | 0.22 | −0.09 | 0.07 |
| Infant biscuits | 0.12 | −0.08 | −0.19 | 0.07 | −0.03 |
| Fish (Fried) | 0.00 | −0.11 | −0.17 | 0.09 | 0.04 |
| Poultry (Fried) | −0.12 | 0.06 | 0.13 | −0.01 | 0.01 |
| Sweet spreads and others | −0.02 | −0.07 | −0.13 | 0.13 | −0.06 |
| Other meats and innards | −0.01 | 0.02 | −0.12 | −0.01 | 0.02 |

***Table S4.*** *Cont.*

| **Food Items** | **Dietary Patterns** | | | | |
| --- | --- | --- | --- | --- | --- |
|  | **Pulses and  Grains** | **Guidelines** | **Predominantly Breastmilk** | **Easy-to-Prepare Foods** | **Noodles (in Soup) and Seafood** |
| White bread | 0.13 | −0.06 | −0.16 | **0.54** | 0.02 |
| Cakes, biscuits, local snacks | −0.13 | 0.06 | 0.09 | **0.52** | 0.03 |
| Clear soup | 0.08 | 0.00 | −0.11 | **0.50** | **0.37** |
| White rice (Plain) | 0.30 | −0.23 | 0.05 | **0.47** | 0.18 |
| Malted drinks | 0.10 | −0.07 | −0.10 | **0.41** | −0.04 |
| Margarine, peanut butter | −0.02 | −0.02 | −0.03 | **0.38** | −0.09 |
| Bread with fillings or toppings | −0.05 | 0.01 | 0.01 | 0.29 | −0.05 |
| Poultry (Low fat) | −0.07 | 0.02 | 0.05 | 0.20 | −0.09 |
| Blended oil | −0.03 | 0.06 | 0.16 | 0.20 | −0.02 |
| Brown rice (Plain) | 0.01 | −0.05 | 0.01 | −0.10 | 0.09 |
| Seafood | −0.01 | −0.02 | −0.01 | −0.11 | **0.65** |
| Noodles (in soup) | −0.10 | 0.03 | −0.08 | 0.08 | **0.63** |
| Cooking oils (Monounsaturated fats) | 0.00 | −0.03 | 0.09 | −0.13 | **0.53** |
| Fresh fruits | 0.12 | **0.25** | 0.08 | −0.01 | **0.37** |
| Eggs | −0.11 | −0.03 | −0.03 | 0.08 | **0.36** |
| Onions, garlic, ginger | 0.00 | −0.03 | **0.26** | −0.05 | **0.32** |
| Dim sum | 0.09 | 0.06 | −0.12 | −0.06 | 0.20 |
| Sweetened drinks | −0.10 | −0.01 | 0.06 | 0.12 | −0.16 |

^a^ Includes sweetcorn, corn starch, millet, buckwheat, barley, ragi flour and semolina powder;
^b^ Includes barley and date water; ^c^ Includes both Indian and Chinese (fried doughstick) fried breads.

**Table S5.** Examples of foods consumed under each food item at 6, 9 and 12 months of age.

| **Food Items** | **Examples** | **Food Items** | **Examples** |
| --- | --- | --- | --- |
| *Beverages* |  | Infant biscuits | Teething rusks, baby bites, puffs |
| Sweetened drinks | Cordial drinks, glucolin water, bandung | *Legumes & pulses* |  |
| Soya bean drinks | - | Bean curds | Soya bean curd, Egg bean curd |
| *Breads* |  | Legumes, lentils | Lentils, red/green beans, kidney beans |
| White bread | - | *Meats* |  |
| Wholemeal breads | Wholegrain, multigrain, softgrain | Pork, beef, lamb (Low fat) | Steamed, boiled, stir fry, stewed |
| Breads with fillings  and/or toppings | Fruit bread, hotdog bun, cream bun | *Milk & dairy* |  |
| Ethnic breads | Chapati, thosai, idli | Malted drinks | Milo, cereal drinks, other malted drinks |
| *Breastmilk* | - | *Noodles & pasta* |  |
| *Confectionary* |  | Noodles (in soup) | Yellow noodles, kway teow, vermicelli |
| Sugar, syrup, condensed milk | Maple syrup, rose syrup | Pasta (in soup) | Macaroni, spaghetti |
| *Desserts & snacks* |  | *Oils & fats* |  |
| Cakes, biscuits, local snacks | Sponge cakes, steamed cakes, marie biscuits | Cooking oil (Unsaturated) | Olive, sunflower, canola oils |
| *Eggs* | - | Margarine, peanut butter | - |
| *Fish & seafood* |  | *Soups & gravies* |  |
| Fish (Low fat) | Steamed, boiled, stir fry, stewed | Clear soup | Pork rib, fish, vegetable soups |
| Seafood | Prawn, scallops, cuttlefish | *Rice & grains alternatives* |  |
| Seafood products,  dried anchovies | Fishball, crabsticks | White rice (Plain) | - |
| *Fruits & juices* |  | Rice porridge | Flavored/Unflavored rice porridge |
| Fresh fruits | Watermelon, apples, pears *etc.* | *Vegetables* |  |

***Table S5.*** *Cont.*

| **Food Items** | **Examples** | **Food Items** | **Examples** |
| --- | --- | --- | --- |
| Fresh juices | 100% fruit juice, infant juice products | Broccoli, cauliflower | - |
| Dried preserved fruits | Raisins, chinese dates, wolfberries | Green leafy vegetables | Spinach, chinese cabbage, celery |
| *Infant products* |  | Yellow, orange, red vegetables | Carrot, tomato, capsicum |
| Infant cereal | - | Starchy vegetables and grouds | White potato, sweet potato, pumpkin |
| Infant formula milk | - | Onions, garlic, ginger | - |

**Table S6.** Correlation coefficients for dietary pattern scores derived from 1-day record and average of 2-day record of the food diaries ^a^.

| **Dietary Patterns** | **6 Months ^b^** | | **9 Months ^c^** | | **12 Months ^d^** | |
| --- | --- | --- | --- | --- | --- | --- |
|  | **Correlation Coefficient** | ***p*-Value** | **Correlation Coefficient** | ***p*-Value** | **Correlation Coefficient** | ***p*-Value** |
| Predominantly breastmilk | 0.802 | <0.001 | 0.520 | <0.001 | 0.820 | <0.001 |
| Guidelines | 0.779 | <0.001 | 0.691 | <0.001 | 0.714 | <0.001 |
| Easy-to-prepare foods | 0.808 | <0.001 | 0.123 | 0.27 | 0.504 | 0.001 |
| Noodles (in soup) and seafood | 0.425 | 0.006 | 0.540 | <0.001 | 0.591 | <0.001 |
| Pulses and grains | - | - | - | - | 0.728 | <0.001 |

^a^ Results were obtained from Pearson correlation coefficient test. Reproducibility and validity of 1-day record from food diaries were assessed with an average of records from 2 other days from the same food diaries; ^b^ Number of subjects used for validation at 6 months = 40; ^c^ Number of subjects used for validation at 9 months = 82; ^d^ Number of subjects used for validation at 12 months = 41.

**Table S7.** Dietary pattern trajectory intercepts and slopes of infants according to characteristics of study sample (*n =* 486) ^a^.

| **Characteristics of Study Sample** | **n (%)** | **Predominantly Breastmilk** | | | | **Guidelines** | | | |
| --- | --- | --- | --- | --- | --- | --- | --- | --- | --- |
|  |  | **Intercept** | ***p*-Value** | **Gradient** | ***p*-Value** | **Intercept** | ***p*-Value** | **Gradient** | ***p*-Value** |
| *Maternal characteristics* |  |  |  |  |  |  |  |  |  |
| **Ethnicity** |  |  | <0.001 |  | <0.001 |  | 0.004 |  | <0.001 |
| Indian | 83 (17.1) | 0.009 ± 0.946 |  | 0.016 ± 0.053 |  | 0.051 ± 0.325 |  | −0.030 ± 0.049 |  |
| Malay | 121 (24.9) | −0.455 ± 0.706 |  | 0.012 ± 0.044 |  | −0.054 ± 0.319 |  | −0.006 ± 0.037 |  |
| Chinese | 282 (58) | 0.198 ± 0.956 |  | −0.010 ± 0.055 |  | 0.008 ± 0.436 |  | 0.012 ± 0.047 |  |
| **Maternal Age** |  |  | 0.001 |  | 0.09 |  | 0.69 |  | 0.34 |
| 18–29 | 206 (42.4) | −0.156 ± 0.903 |  | 0.006 ± 0.054 |  | −0.008 ± 0.345 |  | −0.003 ± 0.044 |  |
| 30–34 | 169 (34.8) | 0.182 ± 0.938 |  | −0.004 ± 0.055 |  | 0.007 ± 0.430 |  | 0.004 ± 0.051 |  |
| >34 | 111 (22.8) | 0.013 ± 0.957 |  | −0.004 ± 0.050 |  | 0.004 ± 0.420 |  | −0.001 ± 0.049 |  |
| **Maternal Education** ^b^ |  |  | <0.001 |  | <0.001 |  | 0.03 |  | 0.09 |
| Primary education | 137 (28.2) | −0.470 ± 0.707 |  | 0.016 ± 0.048 |  | −0.037 ± 0.334 |  | −0.003 ± 0.046 |  |
| Post-secondary | 174 (35.8) | −0.125 ± 0.849 |  | −0.001 ± 0.051 |  | 0.073 ± 0.485 |  | −0.002 ± 0.049 |  |
| University and other | 175 (36.0) | 0.492 ± 0.953 |  | −0.012 ± 0.057 |  | −0.044 ± 0.317 |  | 0.005 ± 0.048 |  |
| **Household Income (SGD)** |  |  | <0.001 |  | 0.001 |  | 0.80 |  | 0.001 |
| <1999 | 62 (12.8) | −0.593 ± 0.603 |  | 0.021 ± 0.043 |  | −0.039 ± 0.297 |  | −0.010 ± 0.043 |  |
| 2000–5999 | 274 (56.4) | −0.078 ± 0.904 |  | −0.004 ± 0.052 |  | 0.006 ± 0.385 |  | −0.003 ± 0.042 |  |
| > 6000 | 150 (30.9) | 0.387 ± 0.951 |  | −0.008 ± 0.058 |  | 0.004 ± 0.441 |  | 0.009 ± 0.058 |  |
| **Employment** |  |  | 0.35 |  | 0.05 |  | 0.84 |  | 0.17 |
| Unemployed | 147 (30.2) | 0.077 ± 1.023 |  | 0.007 ± 0.061 |  | −0.002 ± 0.372 |  | −0.004 ± 0.047 |  |
| Employed | 339 (69.8) | −0.033 ± 0.897 |  | −0.003 ± 0.050 |  | 0.001 ± 0.402 |  | 0.002 ± 0.048 |  |
| **Maternal BMI at 26 weeks** ^c^ |  |  | <0.001 |  | 0.02 |  | 0.09 |  | <0.001 |
| <18.5–24.9 | 219 (45.1) | 0.065 ± 0.961 |  | −0.005 ± 0.056 |  | −0.015 ± 0.417 |  | 0.008 ± 0.049 |  |
| 25.0–29.9 | 180 (37.0) | 0.084 ± 0.935 |  | −0.001 ± 0.052 |  | 0.036 ± 0.419 |  | −0.007 ± 0.048 |  |
| >30.0 | 87 (17.9) | −0.339 ± 0.805 |  | 0.014 ± 0.049 |  | −0.037 ± 0.239 |  | −0.006 ± 0.039 |  |
| **Alcohol** ^d^ |  |  | 0.05 |  | 0.18 |  | 0.55 |  | 0.004 |
| Yes | 171 (35.2) | 0.120 ± 0.976 |  | −0.004 ± 0.055 |  | 0.020 ± 0.458 |  | 0.006 ± 0.050 |  |
| No | 315 (64.8) | −0.065 ± 0.911 |  | 0.002 ± 0.053 |  | −0.011 ± 0.352 |  | −0.003 ± 0.046 |  |
| **Smoking** ^d^ |  |  | <0.001 |  | 0.004 |  | 0.31 |  | 0.68 |
| Yes | 59 (12.1) | −0.503 ± 0.799 |  | 0.015 ± 0.059 |  | −0.011 ± 0.397 |  | −0.005 ± 0.044 |  |
| No | 427 (87.9) | 0.070 ± 0.935 |  | −0.002 ± 0.053 |  | 0.001 ± 0.392 |  | 0.001 ± 0.048 |  |
| **Cohabitation** ^e^ |  |  | 0.39 |  | 0.68 |  | 0.19 |  | 0.11 |
| Not living together | 5 (1.0) | −0.256 ± 1.036 |  | 0.021 ± 0.091 |  | 0.008 ± 0.602 |  | 5.668 × 10^−3^ ± 0.075 |  |
| Living together | 481 (99.0) | 0.003 ± 0.937 |  | −2.218 × 10^−3^ ± 0.053 |  | −8.712 × 10^−4^ ± 0.391 |  | 5.890 × 10^−5^ ± 0.048 |  |
| *Infant characteristics* |  |  |  |  |  |  |  |  |  |
| **Gender** |  |  | 0.22 |  | 0.15 |  | 0.89 |  | 0.24 |
| Female | 232 (47.7) | 0.028 ± 0.895 |  | −0.002 ± 0.050 |  | 4.460 × 10^−3^ ± 0.394 |  | −0.002 ± 0.045 |  |
| Male | 254 (52.3) | −0.025 ± 0.975 |  | 0.002 ± 0.057 |  | −4.074 × 10^−3^ ± 0.393 |  | 0.002 ± 0.050 |  |
| **Parity** |  |  | 0.86 |  | 0.37 |  | 0.78 |  | 0.007 |
| First child | 207 (42.6) | −0.040 ± 0.871 |  | 0.003 ± 0.051 |  | 0.004 ± 0.377 |  | 0.005 ± 0.047 |  |
| Not first child | 279 (57.4) | 0.029 ± 0.984 |  | −0.002 ± 0.055 |  | −0.003 ± 0.405 |  | −0.004 ± 0.048 |  |

Abbreviations: BMI, body mass index; BF, breastfeeding; FM, formula milk. ^a^ Values are means ± SDs or n (%). 2-tailed Mann-Whitney test and Kruskal-Wallis test used to compare the medians between 2 and ≥ 3 groups, respectively; ^b^ Maternal education categorized as primary and secondary education, post-secondary education, as well as university and others; ^c^ Mother’s BMI recorded at 26 weeks of pregnancy (kg/m^2^); ^d^ Statuses recorded prior to pregnancy; ^e^ Reflects the marital status; single, separated or divorced mothers as living separately; married mothers as living together. The ranges of intercepts are: *Predominantly breastmilk* −2.35 to 4.19, *Guidelines* −0.89 to 2.74. The ranges of the gradients are: *Predominantly breastmilk* −0.24 to 0.22, *Guidelines* −0.21 to 0.19.

**Table S8.** Dietary pattern trajectory intercepts and slopes of infants according to characteristics of study sample (*n =* 486) ^a^.

| **Characteristics of Study Sample** | **n (%)** | **Easy-to-Prepare Foods** | | | | **Noodles (in Soup) and Seafood** | | | |
| --- | --- | --- | --- | --- | --- | --- | --- | --- | --- |
|  |  | **Intercept** | ***p*-Value** | **Gradient** | ***p*-Value** | **Intercept** | ***p*-Value** | **Gradient** | ***p*-Value** |
| *Maternal characteristics* |  |  |  |  |  |  |  |  |  |
| **Ethnicity** |  |  | <0.001 |  | 0.25 |  | <0.001 |  | <0.001 |
| Indian | 83 (17.1) | 0.089 ± 0.314 |  | −2.873 × 10^−3^ ± 0.028 |  | 0.021 ± 0.125 |  | −0.007 ± 0.017 |  |
| Malay | 121 (24.9) | 0.003 ± 0.193 |  | 0.006 ± 0.042 |  | 0.022 ± 0.113 |  | −0.008 ± 0.017 |  |
| Chinese | 282 (58) | −0.027 ± 0.211 |  | −0.003 ± 0.025 |  | −0.016 ± 0.187 |  | 0.006 ± 0.034 |  |
| **Maternal Age** |  |  | 0.50 |  | 0.837 |  | <0.001 |  | 0.03 |
| 18–29 | 206 (42.4) | −0.010 ± 0.197 |  | 0.004 ± 0.036 |  | 0.015 ± 0.124 |  | −0.005 ± 0.022 |  |
| 30–34 | 169 (34.8) | −0.016 ± 0.174 |  | −0.001 ± 0.023 |  | 0.003 ± 0.210 |  | 0.002 ± 0.027 |  |
| >34 | 111 (22.8) | 0.043 ± 0.340 |  | −0.005 ± 0.031 |  | −0.032 ± 0.140 |  | 0.005 ± 0.041 |  |
| **Maternal Education** ^b^ |  |  | 0.24 |  | 0.16 |  | 0.58 |  | 0.03 |
| Primary education | 137 (28.2) | −0.003 ± 0.192 |  | 0.006 ± 0.041 |  | 0.005 ± 0.108 |  | −0.004 ± 0.020 |  |
| Post-secondary | 174 (35.8) | 0.004 ± 0.266 |  | 3.095 × 10^−3^ ± 0.028 |  | 0.004 ± 0.194 |  | 0.002 ± 0.037 |  |
| University and other | 175 (36.0) | −0.002 ± 0.224 |  | −0.005 ± 0.023 |  | −0.007 ± 0.166 |  | 0.001 ± 0.025 |  |
| **Household Income (SGD)** |  |  | 0.23 |  | 0.18 |  | 0.03 |  | 0.03 |
| <1999 | 62 (12.8) | 0.031 ± 0.209 |  | 0.005 ± 0.046 |  | −0.020 ± 0.095 |  | −0.002 ± 0.023 |  |
| 2000–5999 | 274 (56.4) | 0.002 ± 0.268 |  | 0.001 ± 0.031 |  | 0.016 ± 0.179 |  | −0.002 ± 0.032 |  |
| > 6000 | 150 (30.9) | −0.017 ± 0.157 |  | −0.004 ± 0.022 |  | −0.021 ± 0.151 |  | 0.004 ± 0.026 |  |
| **Employment** |  |  | 0.53 |  | 0.17 |  | 0.98 |  | 0.52 |
| Unemployed | 147 (30.2) | 0.014 ± 0.277 |  | 0.002 ± 0.038 |  | 0.009 ± 0.194 |  | 0.001 ± 0.029 |  |
| Employed | 339 (69.8) | −0.006 ± 0.209 |  | −0.001 ± 0.028 |  | −0.004 ± 0.147 |  | −0.001 ± 0.029 |  |
| **Maternal BMI at 26 weeks** ^c^ |  |  | 0.09 |  | 0.63 |  | 0.55 |  | 0.11 |
| <18.5–24.9 | 219 (45.1) | −0.008 ± 0.238 |  | −0.002 ± 0.026 |  | −0.014 ± 0.172 |  | 0.003 ± 0.034 |  |
| 25.0–29.9 | 180 (37.0) | 0.006 ± 0.242 |  | 0.0004 ± 0.032 |  | 0.008 ± 0.156 |  | −0.001 ± 0.026 |  |
| >30.0 | 87 (17.9) | 0.006 ± 0.192 |  | 0.005 ± 0.040 |  | 0.017 ± 0.152 |  | −0.005 ± 0.020 |  |
| **Alcohol** ^d^ |  |  | 0.09 |  | 0.29 |  | 0.53 |  | 0.59 |
| Yes | 171 (35.2) | −0.023 ± 0.169 |  | −0.001 ± 0.026 |  | −0.001 ± 0.156 |  | 4.278 × 10^−5^ ± 0.023 |  |
| No | 315 (64.8) | 0.013 ± 0.259 |  | 0.001 ± 0.033 |  | 4.164 × 10^−3^ ± 0.167 |  | −2.330 × 10^−5^ ± 0.032 |  |
| **Smoking** ^d^ |  |  | 0.12 |  | 0.59 |  | 0.03 |  | 0.01 |
| Yes | 59 (12.1) | 0.027 ± 0.246 |  | 0.002 ± 0.032 |  | 0.011 ± 0.110 |  | −0.006 ± 0.021 |  |
| No | 427 (87.9) | −0.004 ± 0.230 |  | −2.872 × 10^−3^ ± 0.031 |  | −0.002 ± 0.169 |  | 0.001 ± 0.030 |  |
| **Cohabitation** ^e^ |  |  | 0.39 |  | 0.68 |  | 0.19 |  | 0.11 |
| Not living together | 5 (1.0) | −0.035 ± 0.188 |  | 0.018 ± 0.055 |  | 0.068 ±0.098 |  | −0.019 ± 0.024 |  |
| Living together | 481 (99.0) | 3.607 × 10^−3^ ± 0.232 |  | −1.823 × 10^−3^ ± 0.031 |  | −7.032 × 10^−3^ ± 0.163 |  | 1.958 × 10^−3^ ± 0.029 |  |
| *Infant characteristics* |  |  |  |  |  |  |  |  |  |
| **Gender** |  |  | 0.05 |  | 0.36 |  | 0.25 |  | 0.62 |
| Female | 232 (47.7) | −0.019 ± 0.195 |  | 0.002 ± 0.032 |  | −0.008 ± 0.129 |  | −4.077 × 10^−3^ ± 0.024 |  |
| Male | 254 (52.3) | 0.018 ± 0.260 |  | −0.001 ± 0.030 |  | 0.007 ± 0.189 |  | 3.723 × 10^−3^ ± 0.033 |  |
| **Parity** |  |  | 0.21 |  | 0.02 |  | 0.04 |  | 0.07 |
| First child | 207 (42.6) | 0.002 ± 0.179 |  | −0.003 ± 0.022 |  | 0.002 ± 0.126 |  | −0.002 ± 0.023 |  |
| Not first child | 279 (57.4) | −0.002 ± 0.264 |  | 0.002 ± 0.036 |  | −0.001 ± 0.186 |  | 0.002 ± 0.033 |  |

Abbreviations: BMI, body mass index; BF, breastfeeding; FM, formula milk. ^a^ Values are means ± SDs or n (%). 2-tailed Mann-Whitney test and Kruskal-Wallis test used to compare the medians between 2 and ≥ 3 groups, respectively; ^b^ Maternal education categorized as primary and secondary education, post-secondary education, as well as university and others; ^c^ Mother’s BMI recorded at 26 weeks of pregnancy (kg/m^2^); ^d^ Statuses recorded prior to pregnancy; ^e^ Reflects the marital status; single, separated or divorced mothers as living separately; married mothers as living together. The ranges of intercepts are: *Easy-to-prepare foods* −0.83 to 2.25, and *Noodles (in soup) and seafood* −0.89 to 1.72. The ranges of the slopes are: *Easy-to-prepare foods* −0.19 to 0.30, and *Noodles (in soup) and seafood* −0.12 to 0.33.

**Table S9.** Associations between “Pulses and grains” dietary pattern scores at 12 months and sociodemographic characteristics (*n* = 486) ^a^.

| **Maternal & Child Characteristics** | **β (95% CI)** | |
| --- | --- | --- |
|  | **Unadjusted Model ^b^** | **Adjusted Model ^c^** |
| *Maternal characteristics* |  |  |
| **Ethnicity** |  |  |
| Indian | 0.815 (0.581, 1.050) *** | 0.815 (0.565, 1.065) *** |
| Malay | 0.006 (−0.197, 0.210) | 0.203 (−0.046, 0.451) |
| Chinese | *Reference* | *Reference* |
| **Maternal Age** |  |  |
| 18–29 | −0.099 (−0.330, 0.133) | −0.152 (−0.390, 0.086) |
| 30–34 | 0.029 (−0.212, 0.269) | −0.070 (−0.300, 0.161) |
| >34 | *Reference* | *Reference* |
| **Maternal Education** ^d^ |  |  |
| Primary education | −0.322 (−0.544, −0.101) ** | −0.200 (−0.474, 0.075) |
| Post-secondary | −0.356 (−0.564, −0.148) ** | −0.235 (−0.468, −0.002) * |
| University and other | *Reference* | *Reference* |
| **Household Income (SGD)** |  |  |
| <1999 | −0.227 (−0.523, 0.069) | −0.124 (−0.473, 0.226) |
| 2000–5999 | −0.201 (−0.400, −0.002) * | −0.134 (−0.364, 0.097) |
| >6000 | *Reference* | *Reference* |
| **Employment** |  |  |
| Unemployed | 0.137 (−0.057, 0.331) | 0.128 (−0.074, 0.330) |
| Employed | *Reference* | *Reference* |
| **Maternal BMI at 26 weeks** ^e^ |  |  |
| <18.5–24.9 | 0.107 (−0.142, 0.356) | 0.159 (−0.092, 0.410) |
| 25.0–29.9 | 0.209 (−0.048, 0.465) | 0.221 (−0.031, 0.472) |
| >30.0 | *Reference* | *Reference* |
| **Alcohol** ^f^ |  |  |
| Yes | −0.209 (−0.395, −0.023) * | −0.110 (−0.310, 0.091) |
| No | *Reference* | *Reference* |
| **Smoking** ^f^ |  |  |
| Yes | −0.264 (−0.536, 0.008) | −0.114 (−0.398, 0.169) |
| No | *Reference* | *Reference* |
| **Cohabitation** ^g^ |  |  |
| Not living together | −0.718 (−1.600, 0.164) | −0.572 (−1.421, 0.277) |
| Living together | *Reference* | *Reference* |
| *Infant characteristics* |  |  |
| **Gender** |  |  |
| Female | −0.125 (−0.303, 0.054) | −0.155 (−0.326, 0.015) |
| Male | *Reference* | *Reference* |
| **Parity** |  |  |
| First child | −0.044 (−0.224, 0.137) | 0.067 (−0.127, 0.261) |
| Not first child | *Reference* | *Reference* |

Abbreviations: BMI, body mass index. ^a^ Results were obtained from general linear models with dietary pattern score as the dependent variable and the sociodemographic covariate(s) as the independent variable(s); ^b^ For the unadjusted model, each sociodemographic covariate was individually associated with the pattern score; ^c^ For the adjusted model, all sociodemographic covariates were the independent variables. Data shown are multivariable linear model β coefficients and their 95% confidence intervals (95% CIs); ^d^ Maternal education categorized as primary and secondary education, post-secondary education, as well as university and others; ^e^ Mother’s BMI recorded at 26 weeks of pregnancy (kg/m^2^); ^f^ Statuses recorded prior to pregnancy; ^g^ Reflects the marital status; single, separated or divorced mothers as living separately; married mothers as living together. * *p*-value < 0.05; ** *p*-value < 0.01; *** *p*-value < 0.001.
